# Mexican Americans With Type 2 Diabetes: Perspectives on Definitions, Motivators, and Programs of Physical Activity

**Published:** 2007-03-15

**Authors:** Nelda Mier, Alvaro A Medina, Marcia G Ory

**Affiliations:** Texas A&M Health Science Center, School of Rural Public Health, South Texas Center; Texas A&M Health Science Center, School of Rural Public Health, South Texas Center; Texas A&M Health Science Center, School of Rural Public Health, South Texas Center

## Abstract

**Introduction:**

Research documents that Mexican Americans bear excess health risk because of physical inactivity and have higher morbidity and mortality rates from chronic diseases than do other ethnic groups. Factors influencing physical activity in this minority population, however, are not well understood. This study examines perceptions of physical activity in a population of Mexican Americans who have type 2 diabetes and live in the Texas-Mexico border region and identifies motivators and barriers to physical activity in this group.

**Methods:**

This study used a qualitative research design and employed six focus groups comprising 39 Mexican Americans with type 2 diabetes who live in the Texas–Mexico border region. A team of bilingual Mexican American researchers systematically reviewed and analyzed focus group data by means of qualitative data analysis software. The study was conducted during 2005–2006.

**Results:**

Most participants considered physical activity to be related not only to exercise but also to occupational and home activities. Walking was the preferred type of activity. Motivators to physical activity included family support and the sense of well-being derived from physical activity. Barriers to physical activity included individual and environmental factors, such as lack of time, physical pain, depression, being overweight, unsafe neighborhoods, and lack of facilities. Participants suggested that the ideal intervention would be low in cost, family-based, close to home, and led by bilingual instructors.

**Conclusion:**

Health promotion efforts to prevent or reduce the effects of chronic disease among Mexican Americans with type 2 diabetes in the Texas–Mexico border region should focus on implementing neighborhood-based, family-oriented walking interventions.

## Introduction

Type 2 diabetes disproportionately affects Hispanics, a rapidly growing population and now the largest minority in the United States. Diabetes prevalence and mortality are two to five times higher in this population than in other ethnic groups (1,2). The prevalence of diabetes in the United States–Mexico border region (15.7%), where the population is predominantly of Mexican descent, is almost twice state (8.3%) and national (9.6%) levels (3,4). A recent study conducted by the Pan American Health Organization reports that the diabetes mortality rate in the Texas–Mexico border region (31.9%) is higher than in the state (24.9%) and in the nation (24.9%) (5).

Some studies suggest that Hispanics have a genetic predisposition to diabetes (6). Nonetheless, the potential of lifestyle changes in diet and physical activity to prevent or delay the onset of type 2 diabetes is well documented (7,8), and studies report low levels of physical activity among Hispanics (9-12). The 2005 Behavioral Risk Factor Surveillance System reports that nationally more Hispanics (56.3%) than non-Hispanic whites (48.9%) fail to meet physical activity recommendations (9). Other studies that include Mexican American women show that this Hispanic subgroup is less likely than any other ethnic subgroup to engage in leisure-time physical activity (10,11). In Texas, more Hispanics (34.5%) than non-Hispanic whites (10.4%) or African Americans (24.8%) are physically inactive. Percentages are higher in the easternmost part of the Texas–Mexico border, where 40.5% of Hispanics and 24% of non-Hispanic whites report high levels of inactivity (12).

Research shows that adults with type 2 diabetes tend to have low levels of physical activity, and national data and research studies indicate that Hispanics with type 2 diabetes are more sedentary than are their counterparts in other ethnic subgroups (13,14). Another study found higher levels of physical inactivity among Mexican Americans with type 2 diabetes (39%) than among non-Hispanic whites (34%) or African Americans with the disease (38%) (15). Despite evidence of the benefits of physical activity, little is known about the motivators and barriers to this behavior among Mexican Americans with type 2 diabetes.

Overall, Mexican Americans account for more than two thirds (66.9%) of Hispanics in the United States. Almost 11 million Mexican Americans are foreign born (39.9%); more than half of these (59.1%) migrated to this country after 1990 (16). Our study, conducted during 2005–2006, examines perceptions of physical activity and identifies motivators and barriers to physical activity among Mexican American adults with type 2 diabetes who live in the Lower Rio Grande Valley (the Valley), a four-county area in the easternmost region of the Texas–Mexico border. This rapidly growing population is closely integrated with Mexico, culturally, socially, and economically (17).

Currently, one million people, almost half the population of South Texas, live in the Valley (18). By 2010, the Valley's population is expected to increase by 27.8%, a higher percentage than in the state (16.0%) and the nation (9.6%) (19,20). Most Valley residents are of Mexican descent (87%); Spanish is spoken in three quarters of homes; 26% of Valley residents are foreign born (compared with 14% statewide and 11% nationally). The Valley fares worse in education, health insurance, and income than do Texas and the nation (18,21,22). Two thirds of individuals younger than 25 years of age have less than a 9th-grade education. Access to medical care is limited, and the major metropolitan areas of the Valley rank last in the nation in per capita income (18,22).

## Methods

This study used a qualitative research design employing six focus groups of Mexican Americans who have type 2 diabetes and live in the Valley. Researchers used a convenience sampling method to select and recruit participants from one of the Valley's four counties. Community-based organizations and community health centers provided referrals. Individuals who agreed to participate were systematically screened for eligibility and had to be 1) aged 30–55 years; 2) diagnosed with type 2 diabetes at least one year before the study (self-reported); 3) without a diagnosis of a cardiovascular disease that would be worsened by exercise and would usually require supervision in a cardiac rehabilitation program; and 4) willing to participate in the study. The Institutional Review Board of Texas A&M University granted permission to conduct the study.

Thirty-nine individuals attended a total of six focus groups. All participants received the same standardized question guide on definitions of physical activity, preferred types of physical activity, motivators and barriers to physical activity, and concepts of culturally sensitive interventions. At the beginning of each focus group, researchers asked participants to read and, if they agreed to participate, to sign a consent form. Researchers read the consent form to some participants with low literacy skills. Focus groups were conducted in English or Spanish, depending on the language preference of participants. Each focus group lasted approximately 60 to 90 minutes, after which participants received a stipend and were personally thanked for their attendance. At the end of the group discussion, participants completed a written questionnaire on their demographic characteristics.

A team of Mexican American graduate students fluent in Spanish and English transcribed focus group discussions from audiotape. Five of the six transcriptions were written in Spanish to maintain the integrity of participants' responses. Only one focus group was conducted in English. Researchers removed all subject identifiers to protect the anonymity of participants and imported the transcripts into QSR N6 (QSR International Pty Ltd, Melbourne, Australia), a software program that codes and manages textual data.

Bilingual Mexican American members of the research team systematically reviewed and coded the transcripts and identified emerging themes. All team members had expertise in border studies related to Mexican Americans and a strong background in physical activity. Researchers based their data analysis on the focus group analysis principles of Morgan and Krueger (23,24). The research team identified, coded, and analyzed key words and emerging themes that indicated participants' issues and concerns. In cases of disagreement about key words or themes during the coding process, the team discussed the issue until reaching a consensus. If no consensus emerged, the principal investigator's decision prevailed.

## Results

Table 1 presents demographic characteristics of the study sample. Of the 39 participants, 34 (87%) were women and 21 (51%) had less than a high school education. A majority of participants were born in Mexico but had lived in the United States for more than 10 years.

### Definition and types of physical activity

Study participants had a broad perspective of the concept of physical activity. Almost all participants viewed physical activity as including not only leisure-time but also work- and home-related activities. Participants also used the terms *physical activity* and *exercise* interchangeably. One participant said that being physically active means "doing housework. When I do housework, I like sweeping and mopping." Another participant stated, "Well, any type of exercise. I heard on the news once that 45 minutes of raking or doing yard work is good, so I do it. It makes you feel better and it helps me, too, so I do it."

Some participants related physical activity to their work activities. One participant stated, "Before I didn't like walking, but when I found out that I had diabetes, I came to the health clinic and I was told that I had to walk. So I started selling Avon products. I go out and walk all around the neighborhood with the Avon products. Also, my little girl is taking dance lessons. In the afternoons, I walk her there and back. I pick her up. I also walk to the nursing home where my dad is. I walk a lot, but I used to be one of those people who did not like to."

Most participants characterized physical activity as housework, yard work, dancing, shopping, and car repair. They also agreed that physical activity includes activities such as jumping, swimming, walking, aerobics, yoga, biking, and running. Most participants, however, preferred walking.

### Motivators and barriers to physical activity

Most participants mentioned that their main motivator for physical activity was the resulting sense of physical and mental well-being. One participant said, "I feel good. It's like I have more energy. I feel more rested, also." Another participant said, "One does it because of the disease [type 2 diabetes] and at the same time to feel better, to look better. The clothing fits better since you are thinner."

Many participants said that family was not only a source of support for physical activity but also a critical motivator because they wanted to be as healthy as possible in order to take care of family members. Someone said, "My wife, she motivates me to get up in the morning, and we both have exercise bikes, but I'm usually the type that says, 'No, I'm not doing that today.' She motivates me to keep going." One woman said that her daughter motivated her to be physically active: "When she wants to play ball or do something else, we all go outside and play catch with her." Another participant said, "We need to be healthy for our grandchildren, too, you know. We need to have that stamina. If we aren't healthy, we can't participate in any activities with them. We're not going to be around."

Almost all focus group participants acknowledged that one of the major barriers to physical activity was not having the time because of work and family obligations. One participant said, "Our dedication is to our families. It's our culture. Women are more prone to be more family-oriented; we're dedicated to our family. We help out more when it comes to our parents, brothers, sisters. I think we are the neutral person in the family." One man added, "At the moment I've been helping my wife with my mother-in-law. She brought her over three weeks ago, and that's kind of what kept me inside the house. It doesn't give me time to do much because we have to watch over her." Other participants agreed that taking care of their children or grandchildren kept them from being physically active. Some participants cited physical pain, depression, lack of motivation, and being overweight as barriers.

All six focus groups mentioned environmental barriers to physical activity. The majority of participants said that unleashed dogs were a major barrier to physical activity and walking in their neighborhoods. One woman said, "With so many dogs around. . . . Oh, man! Those dogs have scared me when going for walks with my boy in the stroller."

For some participants, traffic, lack of sidewalks, poor street lighting, gang activity, and lack of facilities and transportation were environmental barriers. One participant stated, "People are zooming like one hundred miles an hour on the street." Another participant explained, "There are no sidewalks. There are no streetlights. Not even an adequate park. . . . It's true, on the streets, the dogs . . . and we need a park where one can find a safe place to walk." A few participants, however, discounted the poor street lighting and lack of facilities as barriers. One said, "They just built a park in front of my house. I only go across that street and I go for a walk." Another participant said, "Sometimes I go with my husband to the high school. That is where I walk."

For some of the participants, the weather was a barrier to physical activity. One participant said, "Right now the sun is hitting hard, so one rather wants to walk during the evening, but since there is no street lighting, one can't do it." In contrast, another participant stated, "To me the weather does not matter. I am well used to it. I just focus and think, 'It's going to be a hot day,' and our body is made to tolerate the hot weather."

Table 2 summarizes motivators and barriers to physical activity identified by the study sample.

### Physical activity programs

Most participants considered that the ideal physical activity program would have to be low in cost, offered within walking distance of home unless transportation was provided, and led by bilingual, energetic instructors. Participants said that physical activity programs should include walking, weight lifting, dancing, and swimming. One participant said, "I would like for you to come and build a place where you be here some days of the week and ask us to come and walk, do exercise, and even dancing." For some participants, a gymnasium would be the ideal place to become more physically active. One participant stated, "I would be very motivated to go to a gym and exercise there." Some participants qualified this statement, saying that although the town has gymnasiums, they are too expensive or too far away. Most participants agreed that they could afford only a very low fee and would need transportation to get to a physical activity location.

Many participants also thought about an outdoor physical activity program. One participant said, "Some of us can get together somewhere and. . . . Come on! We can encourage and support one another and let's go walking early in the morning. That motivates you a lot. It motivates you a lot — the fact that you are with another person who is supporting you and encouraging you, and at the same time, you are doing the same for that person."

Some participants said that a suitable program should include the entire family. One man stated, "A physical activity program would be very important for us. There are many women with diabetes and many men with diabetes and, unfortunately we, the men, don't come to these programs . . . because the women always say, 'You go and let me stay here,' so it's important to change that and have a program for the entire family that would include the men."

Table 3 summarizes characteristics of an ideal physical activity program suggested by the study sample.

## Discussion

This qualitative study of Mexican American adults who have type 2 diabetes and live in the Valley reveals ways in which this population conceptualizes physical activity, their preferred types of activities, their motivators and barriers to becoming more physically active, and components of their ideal program for promoting physical activity.

Our finding that the study population has a broad perspective of the concept of physical activity resonates with previous research (25,26). Eyler et al (1999) have shown that Hispanic women meet recommendations for physical activity when activities other than those performed during leisure time are examined (27). Other research has shown that Hispanic women engage in significantly more home- and work-related physical activity than do non-Hispanic whites (28).

These results have several research implications. First, further research with Mexican Americans with type 2 diabetes who live on the border with Mexico must emphasize the importance of assessing home- and work-related physical activities. Health benefits associated with these activities should also be examined. Most studies exploring such associations have been conducted mainly with non-Hispanic whites and have been cross-sectional in design, at best (29,30). Second, further research should test the validity and reliability of measurements quantifying moderate and vigorous levels of activity among Mexican Americans living in the border region. In future research, screening tools to identify baseline levels and to track the success of interventions in this population should incorporate an objective, multidimensional scale of physical activity that includes leisure-time, home-, and work-related activities. The use of accelerometers (31,32) could be examined to address the limitations of objective measurements of physical activity in this minority population.

Previous studies support our findings on main motivators for physical activity (33-35). Our findings related to barriers, however, differ from those of previous studies. Whereas earlier studies found that 1) the belief that physical activity is only for the young (34) and 2) not having friends (35) were the main impediments to physical activity, the barriers identified in our study were mostly environmental. This discrepancy may arise from the difference in study groups: previous studies focused on elderly Hispanics or other Hispanic subgroups that did not have type 2 diabetes or did not reside in the Texas–Mexico border region. Another intriguing aspect of our findings is that although a body of quantitative research examines environmental influences on physical activity, no studies with Hispanics have found significant associations between the two (36-39). Our study underlines the relevance of the environment to physical activity in the study population and suggests the need for continued research to determine environmental correlates in the Hispanic subgroup living on the Texas–Mexico border.

Findings on the ideal physical activity program for our study group are consistent with previous studies with elderly Hispanics (34,35). Because walking is the preferred type of physical activity in our sample, an intervention for this population should promote strategies to make walking a lifestyle behavior. Although walking is relatively easy and inexpensive, our study indicates that environmental factors must be addressed to assure the success of any walking program in the border region. The feasibility of, and adherence to, a walking program may depend on having adequate physical and social environments. Policymakers and other health professionals in the border region should advocate not only for programs that promote culturally sensitive interventions but also for policies that make neighborhoods environmentally suitable for physical activity.

Although the generalizability of a qualitative study such as ours is limited, the methodology used can provide insight into a range of meanings offered by participants. As the first study that explores perceptions of physical activity among Mexican Americans with type 2 diabetes who live in the Texas–Mexico border region, this study makes a valuable contribution to the existing literature, despite its limitations. Findings from the study may inform health promotion and policy strategies to prevent or reduce the effects of diabetes among Mexican Americans in the border region by increasing physical activity. This advance could hold the promise of reducing the risks and complications of chronic diseases that are more prevalent in this than in any other ethnic subgroup.

## Figures and Tables

**Table 1:** Demographic Profile of Participants (n = 39), Lower Rio Grande Valley Focus Group Study, Texas, 2005–2006^a^

Characteristic	No. (%)
**Gender **	
Female	34 (87)
Male	5 (13)
**Marital Status **	
Married	30 (77)
Unmarried	9 (23)
**Employment**	
Employed	24 (62)
Unemployed	15 (38)
**Education**	
<High School	21 (54)
<High School	18 (46)
**Country of Birth**	
United States	15 (38)
Mexico	24 (62)
**Years living in the United States^a^**	
0-10	2 (5)
11-15	7 (18)
≥16	27 (69)
**Age, mean (SD)**	34.4 (11.4)

Values are number (percentage) unless otherwise indicated.

a Category does not add to 39 because of missing responses.

**Table 2:** Motivators and Barriers to Physical Activity, Lower Rio Grande Valley Focus Group Study, Texas, 2005–2006

Motivators	Barriers
Sense of physical and mental well-being Family support Staying healthy to take care of the family	Lack of time Physical pain Depression Lack of motivation Being overweight Unleashed dogs Unsafe neighborhood Lack of sidewalks Lack of physical activity facilities Lack of transportation Cost

**Table 3:** Recommended Characteristics of an Ideal Physical Activity Program, Lower Rio Grande Valley Focus Group Study, Texas, 2005–2006

Program Component	Recommended Characteristics
Format and contents	English and Spanish materials Bilingual instructors Materials that are easy to understand and administer Low cost Transportation provided Walking as primary physical activity Create awareness of benefits of physical activity and their association with type 2 diabetes
Interactions	Encouragement of group and family support
Location	Close to participants' homes Adequate setting for targeted physical activity Indoors
